# Voluntary task switching is affected by modality compatibility and preparation

**DOI:** 10.3758/s13421-024-01536-5

**Published:** 2024-02-22

**Authors:** Erik Friedgen, Iring Koch, Edita Poljac, Baptist Liefooghe, Denise Nadine Stephan

**Affiliations:** 1 Institute of Psychology, RWTH Aachen University, Jägerstr. 17/19, D-52066 Aachen, Germany; 2 Radboud University, Postbus 9010, 6500 GL Nijmegen, Netherlands; 3 Utrecht University, Heidelberglaan 1, 3584 CS Utrecht, Netherlands

**Keywords:** Task switching, Voluntary task switching, Modality compatibility

## Abstract

Cognitive task control can be examined in task-switching studies. Performance costs in task switches are usually smaller with compatible stimulus-response modality mappings (visual-manual and auditory-vocal) than with incompatible mappings (visual-vocal and auditory-manual). Modality compatibility describes the modality match of sensory input and of the anticipated response effect (e.g., vocal responses produce auditory effects, so that auditory stimuli are modality-compatible with vocal responses). Fintor et al. (*Psychological Research*, *84*(2), 380–388, 2020) found that modality compatibility also biased task choice rates in voluntary task switching (VTS). In that study, in each trial participants were presented with a visual or auditory spatial stimulus and were free to choose the response modality (manual vs. vocal). In this free-choice task, participants showed a bias to create more modality-compatible than -incompatible mappings. In the present study, we assessed the generality of Fintor et al.’s (2020) findings, using verbal rather than spatial stimuli, and more complex tasks, featuring an increased number of stimulus-response alternatives. Experiment 1 replicated the task-choice bias to preferentially create modality-compatible mappings. We also found a bias to repeat the response modality just performed, and a bias to repeat entire stimulus-response modality mappings. In Experiment 2, we manipulated the response-stimulus interval (RSI) to examine whether more time for proactive cognitive control would help resolve modality-specific crosstalk in this free-choice paradigm. Long RSIs led to a decreased response-modality repetition bias and mapping repetition bias, but the modality-compatibility bias was unaffected. Together, the findings suggest that modality-specific priming of response modality influences task choice.

## Introduction

The processes of cognitive task control can be examined in studies of multitasking. Multitasking research can be subdivided into two major branches: Dual tasking and task switching (see Koch et al., 2018, for a recent review). In both fields, performing two tasks, either simultaneously (dual task) or successively (task switching), leads to performance costs, in form of higher response times (RTs) and error rates, compared to performing just a single task. Moreover, within task-switching blocks, there are additional costs on task-switch trials compared to task-repetition trials, which are known as switch costs (for reviews, see Kiesel et al., 2010; Koch et al., 2018; Monsell, 2003; Vandierendonck et al., 2010). Notably, in task switching, it has been shown in the so-called voluntary task switching (VTS) paradigm that switch costs occur regularly even when participants are given free choice of the task in each given trial, but that there is also a task-repetition bias (e.g., Arrington & Logan, 2004; see Koch & Kiesel, 2022, for a recent review of task switching). The aim of the present study was to examine whether such biases in free choice depend on modality-specific factors. In particular, we sought to assess the generality of the findings of a previous study (Fintor et al., 2020, see below) using different stimuli (verbal instead of spatial), and more complex tasks, with a greater number of stimulus and response alternatives.

In task switching, smaller switch costs have been found when switching between two tasks with modality-compatible mappings (visual-manual and auditory-vocal) than when switching between two tasks with modality-incompatible mappings (visual-vocal and auditory-manual). Modality compatibility describes the match between the modality of the sensory input (visual or auditory) and the modality of the anticipated sensory effect of the response (Stephan & Koch, 2010, 2011): Manual responses usually lead to visible effects, whereas vocal responses lead to auditory effects. As per the ideomotor principle, actions are initiated based on their anticipated effects (James, 1890), so that the anticipation of a visual effect arguably becomes part of the representation of a manual response, and the anticipation of an auditory effect becomes part of the representation of a vocal response. It is notable that modality compatibility, which requires overlap only at the level of the stimulus- and the response-effect modalities, is a less restrictive version of ideomotor compatibility (Greenwald, 1972), which requires overlap even at the level of stimulus and response identity (see Shin et al., 2010, for a review of contemporary ideomotor theory).

One proposed explanation for the modality-compatibility effect on switch costs is increased between-task crosstalk with incompatible modality mappings (Stephan & Koch, 2010, 2011, 2015, 2016). This increased crosstalk is assumed to occur because, with incompatible modality mappings, the anticipated response effect refers to the same input modality as the stimulus of the competing task: In a visual-vocal task, the expected effect of the vocal response is still auditory, which is the stimulus modality of the competing auditory-manual task; and on an auditory-manual trial, the expected effect of the manual response is still visual, which in turn refers to the stimulus modality of the competing visual-vocal task. Because there is no systematic effect of modality compatibility on single-task performance, it is evident that modality-compatibility effects on switch costs cannot be the result of modality-incompatible tasks simply being more difficult overall. Rather, it is the between-task crosstalk that only occurs in mixed-task blocks that seems to be influenced by *combinations* of mappings, specifically (since the sets of stimuli and responses are identical in both modality-compatible and modality-incompatible blocks) (Fintor et al., 2018).

In addition to the ideomotor learning and crosstalk account (Stephan & Koch, 2016), models of multitasking that include modality as a factor (Wickens, 2008), as well as modality-specific accounts of working memory (Baddeley, 1992), can explain these different levels of interference. In particular, the working-memory account suggests that response selection with compatible modality mappings is facilitated because the perceptual and motor codes of each task rely on distinct working-memory subsystems (Maquestiaux et al., 2018). Note that the crosstalk account and the working-memory account are not necessarily mutually exclusive (Friedgen et al., 2021): If both stimulus and response codes refer to the same subsystem, each modality-compatible task would be contained within one subsystem, whereas crosstalk between two subsystems would be present when switching between tasks with modality-incompatible mappings. The crosstalk account by Stephan and Koch (2010, 2011, 2015, 2016) is more specific than the working-memory account, though, since it attributes the linkages between sensory modality and the modality of the anticipated response effect to a specific lifelong ideomotor learning mechanism.

However, modality compatibility does not merely affect multitasking costs in terms of RT and error rates, but also decision biases in voluntary task choice. The advantage of this paradigm is that, contrary to forced-choice modality-compatibility experiments, the crosstalk is not externally caused: Participants are not compelled by the instructions to try and respond in only a modality-compatible manner or only a modality-incompatible manner. Rather, they can create the modality mappings themselves. If they do so based on expected value, as we suggest (Shenhav et al., 2013), a mapping that leads to more modality-specific interference (which has to be resolved before a response can occur) should be less likely to be chosen than a mapping that leads to less modality-specific interference. Indeed, a study by Fintor et al. (2020) demonstrated that, in a VTS paradigm (Arrington & Logan, 2004, 2005), participants chose to form a higher share of compatible modality mappings than of incompatible modality mappings (see Table 1 for an overview of all possible choices of modality combinations). This happened even though participants were asked to use both response modalities (i.e., vocal vs. manual) equally often without resorting to any kind of regularity, suggesting that the observed choice rates represent a bias in voluntary task choice.

**Table 1:** Overview of possible stimulus-response transitions (adapted from Fintor et al., 2020)

Stimulus-response modality mapping		Transition type		
Trial N-1	Trial N	Stimulus modality	Response modality	Stimulus-response mapping
Complete repetition				
** Auditory-vocal**	**Auditory-vocal**	Repetition	Repetition	Repetition
Auditory-manual	Auditory-manual	Repetition	Repetition	Repetition
Visual-vocal	Visual-vocal	Repetition	Repetition	Repetition
** Visual-manual**	**Visual-manual**	Repetition	Repetition	Repetition
Partial switches				
** Auditory-vocal**	Auditory-manual	Repetition	Switch	Switch
Auditory-manual	**Auditory-vocal**	Repetition	Switch	Switch
Visual-vocal	**Visual-manual**	Repetition	Switch	Switch
** Visual-manual**	Visual-vocal	Repetition	Switch	Switch
** Auditory-vocal**	Visual-vocal	Switch	Repetition	Switch
Auditory-manual	**Visual-manual**	Switch	Repetition	Switch
Visual-vocal	**Auditory-vocal**	Switch	Repetition	Switch
** Visual-manual**	Auditory-manual	Switch	Repetition	Switch
Complete switches				
** Auditory-vocal**	**Visual-manual**	Switch	Switch	Switch
Auditory-manual	Visual-vocal	Switch	Switch	Switch
Visual-vocal	Auditory-manual	Switch	Switch	Switch
** Visual-manual**	**Auditory-vocal**	Switch	Switch	Switch

Modality-compatible conditions are shown in bold

Note that VTS research has revealed a general task-repetition bias (Arrington & Logan, 2005; Mittelstädt et al., 2018). Arrington and Logan (2005) explained this task-repetition bias in the form of competing heuristics: A bottom-up process based on (stimulus) availability (Arrington, 2008) can override the top-down mental representation that is trying to generate a random sequence of task choices (see also Mittelstädt et al., 2018). Note that in modality-compatibility studies, a ‘task’ is usually defined as an individual modality mapping (visual-manual, auditory-vocal, visual-vocal, auditory-manual). Accordingly, in the study by Fintor et al. (2020), because stimuli were presented either visually or auditorily, participants could chose only the response modality. Therefore, the task-repetition bias equated a response-modality repetition bias, which was indeed found by Fintor et al. (2020), in addition to the bias to create modality-compatible mappings.

Studies showed that the task-repetition bias is increased by shortening the response-stimulus interval (RSI), which gives participants less time to choose which task to perform once the subsequent stimulus appears (see Arrington et al., 2014, for a review). This effect of RSI on the task-repetition bias has been found irrespective of whether RSI was varied by block (Arrington & Logan, 2004) or by trial (Arrington & Logan, 2005). This suggests that a longer RSI can be used to exert (proactive) cognitive control over the choice bias – and if that is the case, such increased proactive cognitive control may also reduce the modality-compatibility bias on choice rates.

Perseverations like these can also be found with greater response conflict: This triggers a top-down enhancement of the working-memory representation of a task, and then bottom-up processes pick the most activated representation from working memory (Botvinick et al., 2001; Orr et al., 2012). Usually, the most recently activated task set is the most active one, and hence, the task is repeated. But in case modality-compatible mappings have stronger working-memory representations (as suggested by Fintor et al., 2020) – for example, due to both the modality of the stimulus and the anticipated response effect being processed in the same subsystem (Friedgen et al., 2021; Maquestiaux et al., 2018) – this same conflict-monitoring mechanism could also be responsible for biasing choice behaviour in their favour.

Motivation might also play a role, given the costs of exercising cognitive control, thereby incentivising participants to select (modality-compatible) tasks that they find easier (e.g., Shenhav et al., 2013). If the bias to choose a greater share of modality-compatible tasks is but one in-lab example of participants selecting tasks based on expected value, this bias could also be an indicator for how we select tasks in daily life: Everyday tasks that involve crosstalk, modality-specific or otherwise, might be less likely to be chosen voluntarily, because they require the effortful engagement of control processes in order to resolve this crosstalk. This may be the case especially when the payoff for engaging cognitive control is low and/or the penalty for not engaging cognitive control is also low. In a VTS setting, the only ‘reward’ for using control to counteract task-selection biases would be a more comparable frequency of all tasks being chosen, and therefore the intrinsic feeling of having been capable of complying with the instructions (which typically suggests ‘quasi-random’ selection of tasks but still with about equal task frequency). But usually, there is no penalty for participants selecting one task more frequently than another.

Aside from choice rates, RT and error rates remain relevant measures in VTS, too: Task selection and response selection may be different processes (Friedgen et al., 2022), so while task choice is free in VTS, response selection (as in selecting the correct response identity, such as left vs. right) is not. Hence, despite participants selecting the tasks themselves, switch costs are usually still found (for reviews, see Kiesel et al., 2010; Vandierendonck et al., 2010). In fact, Fintor et al. (2020) found even the typical effect of modality compatibility on switch costs (larger switch costs with incompatible modality mappings). By definition, all four mappings (visual-manual, auditory-vocal, visual-vocal, auditory-manual) occurred within each block, since participants could freely choose between a vocal response and a manual response. Hence, Fintor et al. (2020) only compared trials of pure switches (both stimulus-modality and response-modality change) to trials of pure repetitions (both stimulus- and response-modality repeat), not to partial switches (only stimulus-modality or only response-modality changes), as shown in Table 1. This allowed them to approximate the conditions from the previous modality-compatibility studies, in which there had always been fixed mappings of stimulus modality and response modality for each block.

In the present study, we were interested in the influence of modality compatibility on task choice rates in VTS. To this end, we aimed at replicating the basic findings reported by Fintor et al. (2020) with a different experimental setup in Experiment 1. Compared to the study by Fintor et al. (2020), we used verbal task instead of spatial tasks to establish more generality, and we doubled the number of S-R alternatives in each task to increase the difficulty of response selection (four possible stimuli for each stimulus modality, and four possible responses for each response modality). We assumed that the necessity of maintaining a larger stimulus set and a larger response set in working memory would increase cognitive load (Demanet et al., 2010). The conflict between the different options on both the stimulus side and the response side should thus be larger, possibly requiring more cognitive control to resolve. If that is the case, the modality-compatibility effect on choice rates could potentially even be enlarged with this different materials and tasks.

In Experiment 2, we examined whether modality-specific biases in free choice can be reduced if there is more time for cognitive control before the next target stimulus is presented. Because of the nature of a free-choice task, participants could use additional time between trials to decide beforehand which response modality they would like to choose next. Therefore, we varied the RSI and assumed that a longer RSI should allow for more time to exert control over the decision bias by providing more time to prepare for the upcoming choice and thus be less influenced by the modality of the target stimulus. Thus, we predicted smaller modality-specific choice biases with long RSI than with short RSI.

## Experiment 1

### Method

#### Participants

Thirty-two participants (19 female, 29 right-handed, mean age = 24.56 years, SD = 3.307) were tested, eight more than Fintor et al. (2020). In their study, the modality-compatibility effect on RT switch costs had an effect size of η^2^_p_ = .331. According to a power analysis with G*Power 3 (Faul et al., 2007), with alpha = 0.05, a sample size of 30 participants would have been sufficient to find a modality-compatibility effect of this size with a power of 95%. Increasing the sample size to 32 participants raised the power for this effect to over 96%. For the effect of modality compatibility on choice rates, in particular, as stated above, Fintor et al. (2020) found an effect size of η^2^_p_ = .249. The power to find such an effect with the present sample size was over 87%.

All participants had normal or corrected-to-normal vision and hearing and gave their informed consent for participating in the study, receiving either 5 € or partial course credit for their participation.

#### Stimuli and apparatus

The experiment was programmed using PsychoPy2 (Peirce et al., 2019). Stimuli were the visual and auditory number words ‘EINS’, ‘ZWEI’, ‘DREI’ and ‘VIER’ (German for ‘one’, ‘two’, ‘three’ and ‘four’). For visual stimuli we chose number words instead of digits so that stimuli in both modalities would consist of verbal material; this was done for consistency and to avoid confounds between stimulus modality and processing codes (since the auditory stimuli had to be verbal for practical reasons, processing codes for visual stimuli should not be systematically different).

Visual stimuli were presented centrally on a 4:3 screen in white letters on a black background; auditory stimuli were presented binaurally via headphones. Manual responses were made using the middle and index fingers of both hands via four horizontally arranged keys on a wooden board. Vocal responses were made by saying the letters ‘A’, ‘B’, ‘C’ and ‘D’ into a microphone positioned in the centre of the wooden board and in front of the screen.

Both the wooden board and the microphone were connected to the same response box, which was connected to the computer via a USB cable. Because neither the horizontal spatial locations for the manual responses nor the letters for the vocal responses featured direct dimensional overlap with the centrally/binaurally presented numbers one, two, three and four, we assumed that both response modalities would require a comparable amount of sensory-motor transformation. The stimulus ‘one’ required the vocal response ‘A’ or the leftmost manual keypress; the stimulus ‘two’ the vocal response ‘B’ or the centre-left manual keypress; the stimulus ‘three’ the vocal response ‘C’ or the centre-right manual keypress; and the stimulus ‘four’ the vocal response ‘D’ or the rightmost keypress. Participants had a maximum time of 1,500 ms to respond. The RSI was 600 ms, during which the experimenter recorded the accuracy in case the participant chose a vocal response, by pressing the number keys 1–4 on the keyboard (above the letters, not on the number pad, so that all four keys were in one row), in order to convey to the experimental software what the participant had just said (1 = ‘A’, 2 = ‘B’, 3 = ‘C’, 4 = ‘D).^1^ The experimental software could then compare the given response to the required response, just like for manual responses, and then present error feedback when appropriate. Coding vocal responses in this online manner was implemented to enable instant error feedback for *both* response modalities, rather than just measuring response time for vocal responses and determining accuracy in hindsight (accuracy of manual responses was coded automatically from the participants’ keypresses). If the participant made an error, bimodal error feedback (a central red exclamation mark and a ‘boing’ sound) was presented for 400 ms, followed by 100 ms of silence and a black screen, thereby extending the total RSI after an error to 1,100 ms.

#### Procedure

The experiment began with a short practice block of 16 trials in which participants were instructed to try all possible combinations of stimulus and response modalities at least once. After that, six experimental blocks followed. These six experimental blocks consisted of 80 trials with two additional warm-up trials at the beginning of each block (since the first two trials of each block were going to be dropped from the analysis). This added up to 480 experimental trials plus 12 warm-up trials in total. All blocks in this experiment were mixed-task blocks, that is, they featured both visual and auditory (unimodal) stimuli in equal frequency, and participants were asked to respond both manually and vocally. Participants were instructed to vary the response modality randomly while also trying to use both response modalities equally often; to facilitate this, they were told to imagine they were flipping a coin on every trial.

#### Design

The within-subjects independent variables were modality compatibility (incompatible vs. compatible) and switching (switch vs. repetition). The dependent variables were RT, error rates and choice rates. Note that we only manipulated stimulus modality, while modality compatibility and switching were created as a result of the participants’ choices. Hence, modality compatibility was not an independent variable of the experimental setup itself, but rather an independent variable for data analysis. All analyses were conducted at α = .05.

### Results

The practice block and the first two trials of each block were excluded from all analyses. Like in the study by Fintor et al. (2020), for the analysis of RT and error rates, z values for RT were formed for each participant, with all trials outside +/-3 z for a given participant being excluded, as well as all responses following error trials. RT analysis also excluded the error trials themselves.

#### Task choice rate

First, we examined whether participants would form more compatible modality mappings than incompatible modality mappings. The ANOVA on choice rates as a function of modality compatibility revealed a significantly larger proportion of modality-compatible mappings than of modality-incompatible mappings, *F*(1, 31) = 6.505, *p* = .016, η^2^_p_ = .173 (52.5% vs. 47.5%). The more detailed overview over the proportion of choices for the individual stimulus-response modality mappings can be seen in Table 2.

**Table 2:** Mean probability of mapping choices in percent in Experiment 1

	Response modality	
Stimulus modality	Vocal M (SD)	Manual M (SD)
Visual	19.39 (5.24)	**32.64 (5.22)**
Auditory	**19.83 (5.79)**	28.13 (6.10)

Modality-compatible conditions are shown in bold

M = mean, SD = standard deviation

The proportion of manual responses to visual stimuli was significantly higher than to auditory stimuli, *t*(1, 31) = 4.225, *p* < .001. The proportion of vocal responses did not differ significantly between visual and auditory stimuli (*t* < 1)

Next, we ran a *t* test comparing the proportion of vocal responses and manual responses in order to examine whether participants managed to use both response modalities with about equal frequency. This *t* test indicated significantly more manual responses (60.8%) than vocal responses (39.2%), *t*(31) = 6.344, *p* < .001, *d*_*z*_ = 1.121. A second *t* test comparing the proportion of response modality switches and response modality repetitions demonstrated more repetitions (68.8%) than switches (31.2%), *t*(31) = 7.217, *p* < .001, *d*_*z*_ = 1.276, confirming the general response-modality repetition bias.

We also looked at transitions between stimulus-response modality mappings (visual-manual, auditory-vocal, visual-vocal, auditory-manual). For the analysis of choice probability, only a ‘complete’ repetition of both stimulus modality and response modality counted as a repetition, while all full switches (both stimulus modality and response modality switching) and partial switches (either stimulus modality or response modality remaining constant while the other one switches) counted as switches. This means that the expected value of randomness for mapping repetitions was 25%, not 50%. The *t* test indicated a significant upward deviation from 25%, *t*(31) = 9.852, *p* < .001, *d*_*z*_ = 1.742 (38.5% mapping repetitions, 61.5% mapping switches), that is, a mapping repetition bias.^2^

#### Response time and error rates

**Fig. 1:**
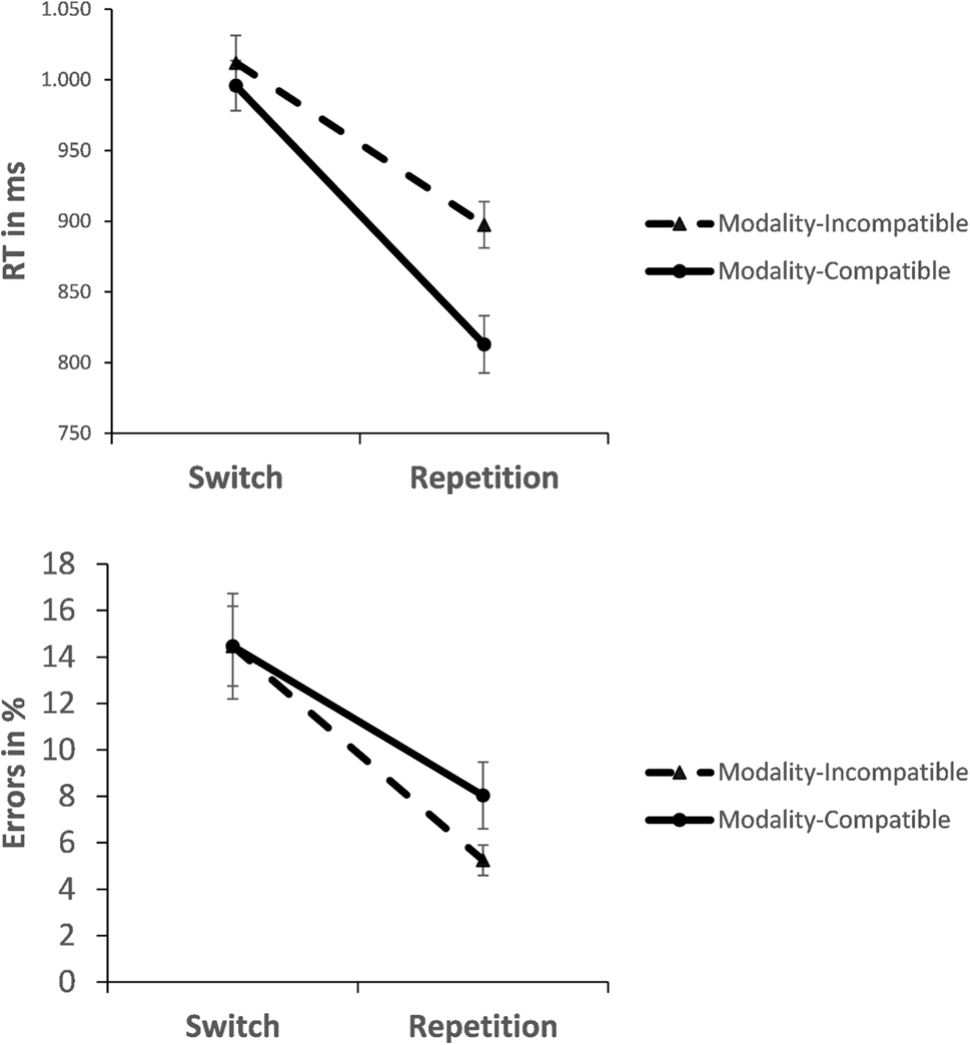
Mean response times (RTs) for the modality-incompatible and the modality-compatible condition on switch trials vs. repetition trials in Experiment 1. A speed-accuracy trade-off can be seen in the repetition condition. Error bars represent the standard error of the mean

For the ANOVA, only complete repetitions of both stimulus modality and response modality counted as repetitions (like visual-manual, visual-manual), and only complete switches of both stimulus modality and response modality counted as switches (like visual-manual, auditory-vocal) (see Fig. 1). As explained before, the theoretically expected share of complete repetitions in case of randomness would have been 25%, and the same is true for complete switches. Given this, it is not surprising that 57.4% of the data qualified for this analysis of complete stimulus-response modality switches and repetitions in RT and error rates (since this is slightly above 50% of the data: the expected share of complete repetitions, about 25%, plus the expected share of complete switches, another 25%). Modality compatibility was coded by collapsing the data across the two modality-compatible and the two modality-incompatible mappings, since modality compatibility is defined by the interaction of stimulus and response modality. Thus, just like in the analysis of choice rates, potential effects of individual stimulus and response modalities were not relevant for our research question.

The 2 × 2 ANOVA for RT with the within-subjects factors modality compatibility (incompatible vs. compatible) and switching (switch vs. repetition) revealed a significant effect of modality compatibility, *F*(1, 31) = 43.100, *p* < .001, η^2^_p_ = .582, with a higher RT for modality-incompatible trials than for modality-compatible trials (955 ms vs. 904 ms). There was also an effect of switching, *F*(1, 31) = 156.762, *p* < .001, η^2^_p_ = .835, confirming a larger RT on switch trials than on repetition trials (1,004 ms vs. 855 ms). Finally, the interaction of modality compatibility and switching was also significant, *F*(1, 31) = 11.868, *p* = .002, η^2^_p_ = .277. However, the direction was inverted relative to the standard effect of modality compatibility on switch costs: Larger switch costs were found in the modality-compatible condition compared to the modality-incompatible condition (183 ms vs. 115 ms). Post hoc *t* tests revealed that this inversion was due to a distinct benefit for modality-compatible mappings in repetition trials, *t*(31) = 7.451, *p* < .001, *d*_*z*_ = 1.317 (897 ms incompatible vs. 813 ms compatible), whereas performance on switch trials did not differ significantly between modality-compatibility conditions, *t*(31) = 1.183, *p* = .246, *d*_*z*_ = .209 (1,012 ms incompatible vs. 996 ms compatible). Note that the error rates show a trend for the interaction in the expected direction, suggesting a speed-accuracy trade-off, as explained below.

In the corresponding ANOVA for error rates, the main effect of modality compatibility was not significant, *F*(1, 31) = 2.716, *p* = .109, η^2^_p_ = .081. The effect of switching was significant, *F*(1, 31) = 30.085, *p* < .001, η^2^_p_ = .493, with more errors on switch trials than on repetition trials (14.5% vs. 6.6%). Finally, the interaction of modality compatibility and switching was not significant, *F*(1, 31) = 1.171, *p* = .288, η^2^_p_ = .036, but the pattern was numerically opposite to that found in RT: Switch costs were higher for the modality-incompatible condition than for the modality-compatible condition (9.3% vs. 6.5%). Post hoc *t* tests showed that performance did not differ between modality-compatibility conditions on switch trials, though, *t*(31) = .002, *p* = .999, *d*_*z*_ = .000 (14.5% in both conditions); rather, there were more errors on modality-compatible repetition trials than on modality-incompatible repetition trials, *t*(31) = 2.129, *p* = .041, *d*_*z*_ = .376 (8.0% vs. 5.2%).

Note that RT and error rates did not differ as a function of modality compatibility in the switch trials, suggesting that there is a specific speed-accuracy trade-off in repetition trials. Since modality-compatibility effects on switch costs can often be found in both RT and error rates, such a pattern of effects trending in opposite directions required further clarification. Hence, to address this trade-off, we performed a post hoc analysis based on inverse efficiency scores (IES), which represent an integrated speed-accuracy measure (Liesefeld & Janczyk, 2019; Vandierendonck, 2017, 2018) by dividing RT in each condition by accuracy (1 – percentage of error rates). In this analysis, there was a significant effect of modality compatibility, *F*(1, 31) = 10.627, *p* = .003, η^2^_p_ = .255 (1,080 ms modality-incompatible vs. 1,037 ms modality-compatible). There was also an effect of switching, *F*(1, 31) = 96.667, *p* < .001, η^2^_p_ = .757 (1,194 ms on switch trials vs. 923 ms on repetition trials). However, using IES, the interaction of modality compatibility and switching was no longer significant (*F* < 1), suggesting that the unexpected direction of the interaction in the RT data can be fully accounted for by a speed-accuracy trade-off in the mapping repetitions.

### Discussion

In Experiment 1, using verbal tasks with four S-R alternatives, we successfully replicated the bias to create more modality-compatible mappings than modality-incompatible mappings. We also found a general mapping-repetition bias. Note, however, that Fintor et al. (2020) had found a mapping-switch bias.^3^ We speculate that this may have been due to increased response conflict in our study, with four alternatives instead of two, leading to a stronger perseveration bias. A stronger perseveration bias may have resulted in participants not only repeating the response modality, but the entire S-R modality mapping. In Experiment 2, we examined whether the observed effects of modality compatibility on voluntary task choice rates would be affected by a variation of the RSI, which represents the time that can be used to exert cognitive control over automatic decision biases.

## Experiment 2

The RSI represents the time between the free response modality choices. As such, it represents an opportunity to engage proactive cognitive control, in order to counteract prepotent response tendencies – including, one would assume, the bias to form a larger share of modality-compatible mappings. A longer RSI should therefore allow participants to form a larger share of modality-incompatible mappings.

Previous studies have shown that the bias to repeat the just-performed task is smaller with long RSIs than with short RSIs (see Arrington et al., 2014, for a review), suggesting a weaker influence of bottom-up processes when participants have more time to choose. Based on the assumption that modality-compatibility effects also reflect bottom-up processes during the response process, we expected them to behave similarly: Hence, Experiment 2 tested whether the bias to form compatible modality mappings can be counteracted with a long RSI compared to a short RSI.

### Method

#### Participants

Thirty-two further participants who had not taken part in Experiment 1 (23 female, 31 right-handed, mean age = 23.31 years, SD = 3.225) were tested. All of them gave their informed consent, receiving the same amount of compensation as the participants in Experiment 1. The power analysis was analogous to Experiment 1, regarding the effect of modality compatibility on switch costs and on choice rates.

#### Stimuli, apparatus and procedure

The experimental setup was the same as in Experiment 1, except for the additional manipulation of the RSI, which was varied randomly trial-by-trial, either being short (400 ms) or long (1,200 ms), compared to the standard RSI of 600 ms we used in Experiment 1. Note that the RSI was the interval in which the experimenter coded vocal responses whenever they occurred. Thus, the RSI needed to be sufficiently long for the experimenter to react; a shorter RSI than 400 ms would not have been possible in a modality-compatibility study with instant error feedback. In the first VTS studies by Arrington and Logan (2004, 2005), which used only manual responses, both the short and the long RSI were shorter, with 100 ms versus 1000 ms, respectively. However, 400 ms has been used as the comparatively shorter RSI in other VTS studies (e.g., Arrington, 2008), and still demonstrated the increased involvement of bottom-up processes*.* The procedure was identical to Experiment 1, again except for the trial-by-trial random variation of the RSI.

#### Design

The independent within-subjects variables for data analyses were modality compatibility (incompatible vs. compatible), switching (switch vs. repetition) and RSI (short vs. long). The dependent variables were RT, error rates and choice proportion. Like in Experiment 1, we only manipulated stimulus modality, and now additionally RSI; modality compatibility and switching resulted from the participants’ choices. All analyses were conducted at α = .05.

### Results

Data analysis for Experiment 2 followed the same procedure as for Experiment 1, only that now, RSI (short vs. long) was an additional factor. Four participants were excluded from the analysis for having error rates larger than 50% in at least one condition, making the final sample size N = 28.

#### Task choice rate

Because participants needed to choose a response modality in each trial regardless of RSI, the RSI could only affect the chosen modality but not whether a response modality is chosen at all (i.e., in VTS research, it is not meaningful to ask for a main effect of RSI on choice rate). Hence, RSI could only affect the influence of other variables on choice rate, and we therefore conducted a series of 2 × 2 ANOVAs to examine potential interactions with RSI on choice rates for modality compatibility, response modality, response modality transition, and mapping transition. Separate ANOVAs were required here, since the factors just listed were different dependent measures that we generated. For example, modality compatibility is defined by the interaction of stimulus modality and response modality; therefore, choice rates of response modality by itself cannot be analysed within the same ANOVA as choice rates of modality compatibility. The overview of choice rates for the individual modality mappings, split up by RSI, can be seen in Table 3.

**Table 3:** Mean probability of mapping choices in percent in Experiment 2

	Response modality			
	RSI = 400 ms		RSI = 1200 ms	
Stimulus modality	Vocal M (SD)	Manual M (SD)	Vocal M (SD)	Manual M (SD)
Visual	19.08 (6.1)	**32.23 (6.8)**	19.32 (5.9)	**32.87 (4.9)**
Auditory	**20.63 (6.5)**	28.06 (6.3)	**19.54 (6.5)**	28.27 (7.5)

Modality-compatible conditions are shown in bold. The proportion of manual responses to visual stimuli was significantly higher than to auditory stimuli, *t*(1, 27) = 4.102, *p* < .001. The proportion of vocal responses did not differ significantly between visual and auditory stimuli (*t* < 1), but numerically, there were more vocal responses to auditory than to visual stimuli. The 3-way interaction of stimulus modality, response modality, and RSI on choice rates was not significant (*F* < 1)

To examine modality compatibility, the ANOVA with modality compatibility and RSI as independent variables yielded a significant effect of modality compatibility on choice rates, *F*(1, 27) = 6.714, *p* = .015, η^2^_p_ = .199, confirming a higher share of modality-compatible mappings than of modality-incompatible mappings (52.6% vs. 47.4%). However, this effect was not further modulated by RSI (*F <* 1). A follow-up Bayesian analysis for the interaction of modality compatibility and RSI revealed a *BF*_10_ = 0.29, which can be seen as substantial evidence for the null hypothesis (Jeffreys, 1961), that is, no difference in the size of the modality-compatibility effect on choice rates depending on RSI.

To examine response-modality preference, we ran an ANOVA on choice rate with response modality and RSI as independent variables. This yielded an effect of response modality, *F*(1, 27) = 28.832, *p* < .001, η^2^_p_ = .516, with more manual responses than vocal responses (60.7% vs. 39.3%). This effect was not modulated by RSI either.

The ANOVA for response modality transition and RSI confirmed the response-modality repetition bias, *F*(1, 27) = 78.033, *p* < .001, η^2^_p_ = .743 (70.5% repetitions vs. 29.5% switches). RSI interacted with response modality transition, *F*(1, 27) = 42.167, *p* < .001, η^2^_p_ = .610, indicating the novel finding of a stronger response-modality repetition bias after the short RSI (72.8% repetitions, 27.2% switches) than after the long RSI (68.2% repetitions vs. 31.8% switches).

To examine the mapping switch versus repetition bias as in Experiment 1, the ANOVA for mapping transition and RSI yielded an effect of mapping transition, *F*(1, 27) = 103.903, *p* < .001, η^2^_p_ = .794, with more mapping switches than mapping repetitions overall (61.4% vs. 38.6%). However, keep in mind the decisive threshold to distinguish a mapping switch bias from a mapping repetition bias was the expected value of randomness for repetitions, 25%: The *t* test confirmed the overall rate of mapping repetitions (38.6%) was significantly higher than 25%, *t*(27) = 12.170, *p* < .001, *d*_*z*_ = 2.300, indicating a mapping repetition bias, consistent with Experiment 1. Again, there was no main effect of RSI (*F* < 1). However, the interaction of mapping transition and RSI was significant, *F*(1, 27) = 13.348, *p* = .001, η^2^_p_ = .331, showing a greater share of mapping repetitions after a short RSI (40.1% repetitions, 59.9% switches) than after a long RSI (37.1% repetitions, 62.9% switches). Post hoc *t* tests confirmed this second novel finding, with a larger effect size for the mapping repetition bias after the short RSI, *t*(27) = 14.043, *p* < .001, *d*_*z*_ = 2.654, than after the long RSI, *t*(27) = 9.324, *p* < .001, *d*_*z*_ = 1.762.

#### Response time and error rates

**Fig. 2:**
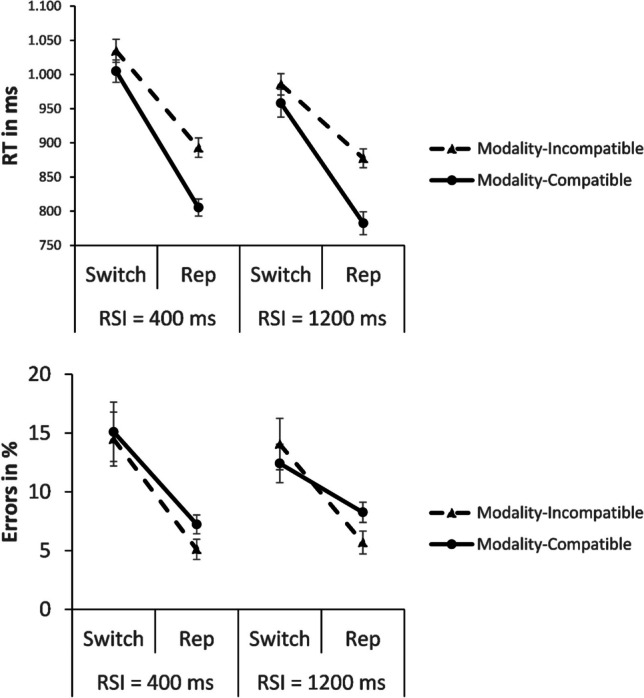
Mean response times (RTs) and error rates for the modality-incompatible and the modality-compatible condition on switch trials vs. repetition trials (Rep) in Experiment 2, split up by RSI (400 ms = short; 1,200 ms = long). Error bars represent the standard error of the mean

Because RT (and error rates) could be affected directly by RSI, we conducted a full-factorial 2 × 2 × 2 ANOVA (see Fig. 2) for RT with the within-subjects independent variables modality compatibility (incompatible vs. compatible), switching (switch vs. repetition), and RSI (short vs. long). This ANOVA yielded a significant effect of modality compatibility, *F*(1, 27) = 47.039, *p* < .001, η^2^_p_ = .635, with overall slower responses on modality-incompatible trials than on modality-compatible trials (948 ms vs. 888 ms). There was also an effect of switching, *F*(1, 27) = 200.372, *p* < .001, η^2^_p_ = .881, showing a higher RT on switch trials compared to repetition trials (996 ms vs. 840 ms).

The main effect of RSI was in fact significant, too, *F*(1, 27) = 22.462, *p* < .001, η^2^_p_ = .454, with a shorter RT after a long RSI than after a short RSI (901 ms vs. 935 ms). RSI yielded a trend for an interaction with switching, *F*(1, 27) = 3.372, *p* = .077, η^2^_p_ = .111, showing numerically smaller switch costs after a long RSI than after a short RSI (142 ms vs. 171 ms). But neither the two-way interaction of modality compatibility and RSI (*F* < 1), nor the three-way interaction of modality compatibility, switching, and RSI was significant (*F* < 1).

Yet, the interaction of modality compatibility and switching was significant, *F*(1, 27) = 11.981, *p* = .002, η^2^_p_ = .307. Consistent with Experiment 1, the interaction effect showed the unexpected pattern, with larger switch costs on modality-compatible trials than on modality-incompatible trials (187 ms vs. 125 ms). Again, post hoc *t* tests suggested this inversion was based on a particular 91-ms advantage for modality-compatible repetition trials over modality-incompatible repetition trials, *t*(27) = 7.712, *p* < .001, *d*_*z*_ = 1.457 (885 ms modality-incompatible vs. 794 ms modality-compatible), whereas the performance difference on switch trials was 29 ms smaller between modality-compatibility conditions, but still significant, *t*(27) = 2.156, *p* = .040, *d*_*z*_ = .407 (1,010 ms modality-incompatible vs. 981 ms modality-compatible).

In the ANOVA for error rates, there was no main effect of modality compatibility, *F*(1, 27) = 1.215, *p* = .280, η^2^_p_ = .043. The effect of switching was significant, *F*(1, 27) = 31.740, *p* < .001, η^2^_p_ = .540, showing more errors on switch trials than on repetition trials (14.0% vs. 6.6%). There was no effect of RSI (*F* < 1), and there were no interactions with RSI: neither between RSI and switching (*F* < 1), nor between RSI and modality compatibility (*F* < 1), nor the three-way interaction of modality compatibility, switching, and RSI (*F* < 1).

The interaction of modality compatibility and switching was not significant, *F*(1, 27) = 1.641, *p* = .211, η^2^_p_ = .057. But like in Experiment 1, in contrast to RT analysis, for errors the trend went into the expected direction, with numerically larger switch costs on modality-incompatible trials than on modality-compatible trials (8.9% vs. 6%). Therefore, different directions of the influence of modality compatibility on switch costs in RT and error rates again suggest a speed-accuracy trade-off. To account for this trade-off, we again conducted a post hoc analysis of IES. In this analysis of IES, the main effect of modality compatibility was significant, *F*(1,27) = 18.642, *p* < .001, η^2^_p_ = .408 (1,249 ms incompatible vs. 1,152 ms compatible). The effect of switching was also significant, *F*(1, 27) = 137.309, *p* < .001, η^2^_p_ = .836 (1,503 ms on switch trials vs. 898 ms on repetition trials). There was also the effect of RSI, *F*(1,27) = 5.771, *p* = .023, η^2^_p_ = .176, with higher IES after the short RSI than after the long RSI (1,255 ms vs. 1,146 ms). However, the interaction of modality compatibility and switching was not significant (*F* < 1), nor was the interaction of modality compatibility and RSI (*F* < 1), nor the three-way interaction of modality compatibility, switching, and RSI (*F* < 1). Hence, when taking the speed-accuracy trade-off into account by calculating IES, the surprising interaction pattern in RT disappeared.

### Discussion

In Experiment 2, we varied the RSI (400 ms vs. 1,200 ms) randomly from trial to trial. While the very first VTS study manipulated RSI by block (Arrington & Logan, 2004), the effect of a weaker task-repetition bias with long RSIs has also been found with trial-wise manipulation (Arrington & Logan, 2005). With the latter type of manipulation, we replicated this influence of RSI on the task-repetition bias (response-modality repetition bias in our case) and also found such an RSI influence on the mapping-repetition bias: Both types of repetition biases were less pronounced after long RSIs. However, there was no additional influence of RSI on the proportion of modality-compatible mappings formed, with substantial evidence from the Bayes factor supporting the absence of the effect.

Yet, independent from the RSI, we confirmed the novel mapping-repetition bias first found in Experiment 1. Also, we once more replicated both the response-modality repetition bias and the bias to form a greater share of compatible modality mappings, consistent with Fintor et al. (2020).

Note that, using block-wise instructed modality mappings (e.g., visual-manual and auditory-vocal for a modality-compatible block) instead of voluntary task choice, Stephan and Koch (2010) found that that the influence of modality compatibility on switching was smaller with longer RSI, whereas we did not find this specific RSI influence. Many factors might explain this difference across studies. One factor is the range of RSIs used: They used generally longer RSIs (600 ms vs. 1,600 ms compared to 400 ms vs. 1,200 ms in the present study). Probably more importantly, this difference might also be due to the general difference between instructed and voluntary task choice. Finally, unlike in Stephan and Koch’s (2010) study, in the present study all four possible S-R modality combinations (visual-manual, auditory-vocal; visual-vocal, auditory-manual) were always featured together within the same blocks – whereas in forced-choice modality compatibility studies, entire blocks containing only modality-compatible mappings are compared against entire blocks containing only modality-incompatible mappings. Hence, the role of RSI might also be reduced under those conditions, which occurred in the present study as well.

## General discussion

In two experiments, we examined the effects of modality compatibility in VTS. We found evidence for a bias to form more modality-compatible mappings in both experiments. This effect was about the same size as found by Fintor et al. (2020). Compared to that earlier study, in both experiments, we found a mapping-repetition bias as opposed to a mapping-switch bias, and a preference for manual responses, that is, manual dominance rather than vocal dominance. As expected, we also found a bias to repeat the just-performed response modality, which in our design represents a task-repetition bias (Arrington & Logan, 2004, 2005; Arrington et al., 2014; Mittelstädt et al., 2018). Both the task-repetition bias and the mapping-repetition bias were stronger after a short RSI. In contrast, crucially, the decision bias to form a greater share of modality-compatible mappings was *not* significantly reduced with longer time before the choice, that is, after a long RSI.

The fact that we have twice replicated Fintor et al. (2020)’s modality-compatibility bias on choice rates supports our proposition of participants selecting tasks based on expected internal value, in terms of effort of control (Shenhav et al., 2013). The absence of an interaction with RSI suggests that additional time to prepare the task choice does not generally suffice for participants to override this tendency. This is striking, given that RSI did influence several other effects of both RT and choice rates, as we will discuss in the following.

### The influence of RSI

The long RSI led to faster responses overall, suggesting that our manipulation was indeed effective, due to more time for preparing the next choice (in case any preparation occurred), as well as more time for potential decay of the working-memory representation of the previous choice (Koch & Kiesel, 2022; Meiran, 1996). In line with previous VTS studies (e.g., Arrington & Logan, 2004, 2005; Mittelstädt et al., 2018), the duration of the RSI further influenced choice rates – however, not with regards to the share of modality-compatible mappings formed, but concerning the rate at which response modalities and stimulus-response mappings were repeated. This suggests the modality-compatibility bias on choice rates is different in nature from both our present response-modality repetition bias and the task-repetition bias more generally.

The task-repetition bias is assumed to arise because bottom-up processes select the most active task representation from working memory (Botvinick et al., 2001; Orr et al., 2012); overcoming this perseveration bias consequently requires engaging top-down processes (Arrington & Logan, 2005; Mayr & Bell, 2006). Since in modality-compatibility studies, we define ‘task’ as synonymous with ‘modality mapping’, this explanation for the task-repetition bias holds true for both the response-modality repetition bias and the mapping-repetition bias. Participants could of course only select the response modality, but in doing so, they formed compatible or incompatible S-R modality mappings as a result. One potential cause of the mapping-repetition bias in our present study, as opposed to the mapping-switch bias reported by Fintor et al. (2020), might be the presumed greater response conflict with our setup: Having four instead of two alternatives for each stimulus modality and each response modality requires the maintenance of larger task sets (that is, more numerous response alternatives) in working memory.

These task-repetition biases get smaller with a long RSI because, presumably, either there is more time to reconfigure the task set if the participant is intending a switch (Arrington & Logan, 2004; Rogers & Monsell, 1995) or because there is more time to allow the persisting activation of the previous task set to decay (Allport et al., 1994; see Vandierendonck et al., 2010, for a review). Note that, because response modality is by definition predictable in free-choice paradigms, proactive control seems to be a more plausible influence; decay merely cannot be ruled out entirely, as an additional explanation. In either case: With a long RSI, there is more time to resolve the conflict between the current and the previous task set. This would make the bottom-up processes less likely to pick the most active task representation from working memory. Conversely, with a short RSI, there should be less time to engage top-down processes prior to stimulus onset to overcome not only the perseveration bias, but also the bias to form more modality-compatible mappings, hence resulting in a larger decision bias.

Yet, since the share of compatible modality mappings did not differ between short and long RSIs, this is not in line with the suggestion by Fintor et al. (2020) that modality-compatible mappings have stronger working-memory representations in general. Even though, with compatible modality mappings, both the stimulus modality and the modality of the anticipated response effect refer to the same working-memory subsystem (Friedgen et al., 2021; Maquestiaux et al., 2018), this would then not imply locally stronger working-memory representations of those mappings within the respective subsystem – meaning, the phonological loop or visuospatial sketchpad (Baddeley, 1992).

Instead, if there is no such locally stronger working-memory representation creating a performance benefit for compatible modality mappings, the issue might instead lie with incompatible modality mappings being represented in both subsystems – specifically, with *both* modality-incompatible tasks partially referring to *both* subsystems, so that the modalities of the stimuli and the anticipated response effects overlap *between* subsystems, rather than within. In short: If the present results can be interpreted as evidence against stronger working-memory representations of modality-compatible mappings, this instead lends further credence to the crosstalk account (Friedgen et al., 2021; Schacherer & Hazeltine, 2020, 2021; Stephan & Koch, 2010, 2011). That is, modality-compatibility effects are attributed to greater between-task interference with incompatible modality mappings.

That said, note that we cannot rule out the possibility that modality-compatibility effects on choice rates might still be larger if the RSI is even shorter than 400 ms – for example, in the studies by Arrington and Logan (2004, 2005), the short RSI was only 100 ms long. A minimum RSI duration of 400 ms was required in our experiment, though, to enable the experimenter to code the participant’s vocal responses and thereby provide instant error feedback. Even though in VTS, only errors of response identity are possible, not errors of task choice (which are usually the more common type of error in forced-choice modality-compatibility experiments), our paradigm still required this instant error feedback – among others, because of the increased number of S-R alternatives. Hence, with our somewhat longer short RSI compared to studies using manual responses, we might be underestimating the role of active preparation during the RSI because some of it might have occurred already during the short RSI.

When speaking of preparation in the context of the present VTS experiments, remember that only the upcoming response modality could be prepared (chosen) during the RSI. Some recent studies in dual tasking suggested that the central response-selection bottleneck (e.g., Pashler, 1994) can be bypassed via preparation, by loading the stimulus-response mappings into working memory (Lyphout-Spitz et al., 2022; Maquestiaux et al., 2020). In our present study, though, both stimulus modality and the required response identity (based on the numerical characteristics of the stimuli) could not be known before stimulus onset. A participant could therefore only decide to repeat or switch the response modality (referring to the task-repetition bias); but they could not decide to repeat or switch the entire stimulus-response modality mapping (referring to the mapping-repetition bias we found, or the mapping-switch bias found by Fintor at al., 2020). Hence, pre-loading the entire stimulus-response modality mapping into working memory should not have been possible with our present setup. This would serve as another explanation for why the task-repetition bias was indeed increased after short RSIs (less time to load the other response modality into working memory, to replace the one that had just been used), but the modality-compatibility bias on choice rates was not increased after short RSIs. However, this account does not explain why the mapping-repetition bias was also increased after short RSIs, since the decision of repeating or switching the response modality on the upcoming trial, at least during the RSI that precedes it, cannot be made in the context of knowing the stimulus modality.

#### Manual dominance

The finding of manual dominance – relative to vocal dominance in Fintor et al. (2020) – could be explained by an easier sensory-motor transformation from the number stimuli to four horizontally aligned keys, going along with the mental number line from left to right, compared to the transformation required from number words to the spoken letters. Thus, numerical-spatial associations may produce SNARC-like compatibility (Dehaene et al., 1993) that might have created stronger dimensional overlap (Kornblum et al., 1990) than the numerical-alphabet associations for the vocal responses. Moreover, the fact that both experiments in the present study were conducted during the COVID-19 pandemic, with participants being required to wear masks in the lab, may also have contributed to this shift in preferred response modality; Fintor et al. (2020) attributed vocal dominance in their study specifically to the effort involved in producing vocal responses, and the masks may have increased this physical effort in our case. Note though that this general preference for one respective response modality, in both our study and theirs, cannot explain how choice rate is influenced by modality compatibility, which is defined by the interaction of stimulus modality and response modality, and is thus independent of the main effect of modality (i.e., vocal vs. manual dominance bias).

#### Speed-accuracy trade-off

In both experiments, the effect of modality compatibility on switch costs was reversed in RT, but the analysis of IES indicated a speed-accuracy trade-off, which, if taken into account, no longer revealed an effect of modality compatibility on switch costs. Note that the trade-off was restricted to the mapping-repetition condition, suggesting no generally better performance with modality-compatible conditions. This suggests the possibility of an adjustment of response threshold (= a lower response threshold when repeating modality-compatible mappings, resulting in faster responses at the cost of accuracy). This hypothetical threshold adjustment was not evident in Fintor et al. (2020).

The trade-off could be due to the differences with regard to forced-choice modality-compatibility studies with fixed modality mappings: The free task choice allowed participants to switch between all four possible mappings (visual-manual, auditory-vocal, visual-vocal, auditory-manual) within the same block, rather than just between two compatible or two incompatible modality mappings within the same block. Even though, in line with previous research (Fintor et al., 2018), the ANOVA only compared switches within modality-compatibility conditions, not between modality-compatibility conditions (e.g., not a switch from visual-manual to visual-vocal), this exclusion of partial switch trials (for an analysis, see Appendix) of course reduced the overall number of switch trials that could be contrasted in the analysis of RT and error rates. A different explanation for the unclear modality-compatibility effect on RT and error switch costs is the lower conceptual overlap (Schacherer & Hazeltine, 2019, 2021) between stimuli and responses in our present study compared to the strong spatial compatibility and thus dimensional overlap in Fintor et al. (2020)’s setup: Schacherer and Hazeltine (2019) demonstrated that reducing conceptual overlap in both tasks can make the modality-compatibility effect on switch costs disappear.

Finally, while there is also the difference between our study using verbal stimuli versus Fintor et al.’s (2020) study using spatial stimuli, we do not think this difference is behind the speed-accuracy trade-off occurring in our study while being absent in Fintor et al.’s (2020): In our previous study comparing verbal processing codes versus spatial processing codes in forced-choice task switching (Friedgen et al., 2021), a speed-accuracy trade-off was also found, but with the spatial stimuli; meanwhile, with verbal processing codes, the modality-compatibility effect on switch costs pointed in the expected direction (larger switch costs in the modality-incompatible condition). Therefore, the use of verbal processing codes in the present VTS study cannot account for the current speed-accuracy trade-off in Experiments 1 and 2. In any case: This trade-off should not detract from the main findings, which refer to the choice rates in VTS.

## Conclusion

This study examined modality-specific factors that can exert a bias in task decision processes, and we examined whether this bias is influenced by the time between task choices (i.e., the RSI). Specifically, using voluntary task switching, we found that participants created more modality-compatible mappings than modality-incompatible mappings if given free task choice. They were also more likely to repeat not only response modalities, but entire stimulus-response modality mappings – especially so after short RSIs, even though the specific bias to prefer modality compatibility was not affected by RSI. These findings extend previous research by showing that modality compatibility affects voluntary task choices, but not in the same way that perseveration biases do. The modality-compatibility bias seems to be of a different kind than other task-selection biases – one that might not be as sensitive to prolonged time for engaging proactive cognitive control. Hence, in contrast to perseveration biases, we attribute modality-compatibility effects on choice rates to greater between-task crosstalk with incompatible modality mappings.

## Appendix

### Analysis of partial switches/partial repetitions

We performed additional analyses on RT and error rates for partial transitions (either only stimulus modality repeated but response modality switched, or only response modality repeated but stimulus modality switched). By design, all of these partial switches were switches between modality-compatible and incompatible conditions (e.g., visual-manual, visual-vocal; or auditory-vocal, visual-vocal). We contrasted these against full repetitions of both stimulus modality and response modality on the one hand; and against full switches of both stimulus modality and response modality on the other hand.

In Experiment 1, the two-way ANOVA with the independent within-subjects variables modality compatibility (incompatible vs. compatible) and switching (partial switch vs. full repetition) yielded an effect of modality compatibility in RT (945 ms compatible vs. 852 ms incompatible), *F*(1, 31) = 94.371, *p* < .001, η^2^_p_ = .753, and reversed in error rates (7.0% incompatible vs. 9.1% compatible), *F*(1, 31) = 5.258, *p* = .029, η^2^_p_ = .145. There was also an effect of partial switching in RT (941 ms on partial switch trials vs. 855 ms on full repetition trials), *F*(1, 31) = 109.613, *p* < .001, η^2^_p_ = .780, which was consistent in error rates (9.5% on partial switch trial vs. 6.6% on full repetition trials), *F*(1, 31) = 14.011, *p* = .001, η^2^_p_ = .311. The interaction of modality compatibility and partial switching was neither significant for RT, nor for error rates (both *p*s > .10).

The two-way ANOVA with the independent within-subjects variables modality compatibility (incompatible vs. compatible) and switching (partial switch vs. full switch) yielded an effect of modality compatibility in RT (1002 ms incompatible vs. 943 ms compatible), *F*(1, 31) = 49.860, *p* < .001, η^2^_p_ = .617, but not in error rates (*F* < 1). There was also an effect of partial switching, with worse performance on full switch trials (1,004 ms / 14.5%) than on partial switch trials (941 ms / 9.5%)), both in RT, *F*(1, 31) = 39.371, *p* < .001, η^2^_p_ = .559, and in error rates, *F*(1, 31) = 15.506, *p* < .001, η^2^_p_ = .333. Finally, the interaction of modality compatibility and switching was significant in RT, *F*(1, 31) = 21.149, *p* < .001, η^2^_p_ = .406, with larger costs for full switches compared to partial switches in the modality-compatible condition (105 ms) than in the modality-incompatible condition (20 ms). In error rates, this interaction was not significant (*F* < 1).

In Experiment 2, the ANOVAs additionally included the independent within-subjects variable RSI (short vs. long), and as outlined in the main text, four subjects had to be excluded due to low accuracy of their performance. The two-way ANOVA with the independent within-subjects variables modality compatibility (incompatible vs. compatible), RSI (short vs. long), and switching (partial switch vs. full repetition) yielded an effect of modality compatibility in RT (929 ms incompatible vs. 838 compatible), *F*(1, 27) = 73.935, *p* < .001, η^2^_p_ = .733; in error rates, there was a non-significant trend in the opposite direction (7.0% incompatible vs. 8.2% compatible), *F*(1, 27) = 3.131, *p* = .088, η^2^_p_ = .104. There was also an effect of partial switching, both in RT (927 ms on partial switch trials versus 840 ms on full repetition trials), *F*(1, 27) = 269.592, *p* < .001, η^2^_p_ = .909, and in error rates (8.5% on partial switch trials vs. 6.6% on full repetition trials), *F*(1, 27) = 14.213, *p* = .001, η^2^_p_ = .345. The effect of RSI was significant in RT (897 ms after short RSIs versus 870 ms after long RSIs), *F*(1, 27) = 17.577, *p* < .001, η^2^_p_ = .394, but not in error rates (*F* < 1). Only in error rates, there was an interaction of modality compatibility and partial switching, *F*(1, 27) = 5.313, *p* = .029, η^2^_p_ = .164, with larger partial switch costs in the modality-incompatible condition (3.1%) than in the modality-compatible condition (0.8%). All other effects were non-significant (all *p*s > .20).

The ANOVA with the independent within-subjects variables modality compatibility (incompatible vs. compatible), RSI (short vs. long), and switching (partial switch vs. full switch) yielded an effect of modality compatibility in RT (992 ms incompatible vs. 931 ms compatible), *F*(1, 27) = 46.965, *p* < .001, η^2^_p_ = .635, but not in error rates (*F* < 1). There was also an effect of partial switching, with worse performance on full switch trials than on partial switch trials, both in RT (996 ms vs. 927 ms), *F*(1, 27) = 37.599, *p* < .001, η^2^_p_ = .582, and in error rates (14.0% vs. 8.5%), *F*(1, 27) = 15.950, *p* < .001, η^2^_p_ = .371. The effect of RSI was significant in RT (983 ms after short RSIs vs. 941 ms after long RSIs), *F*(1, 27) = 25.128, *p* < .001, η^2^_p_ = .482, but not in error rates (*F* < 1). Finally, the interaction of modality compatibility and partial switching was significant in RT, *F*(1, 27) = 13.095, *p* = .001, η^2^_p_ = .327, but not in error rates (*F* < 1), with larger costs on full switch trials compared to partial switch trials in the modality-compatible condition (100 ms) than in the modality-incompatible condition (36 ms). All other effects were non-significant (all *F*s < 1).

## Footnotes

1 The program did not wait for the experimenter’s response, but always waited for the duration of the RSI (600 ms in Experiment 1; 400 ms vs. 1,200 ms in Experiment 2). If the experimenter entered the wrong response, or failed to respond within the specified time interval, the entered response did not match the response required on this trial – therefore, such trials were treated as errors. Based on our experience during testing, both in this study and in previous experiments using the same setup for online coding of vocal responses, an error on the experimenter’s part did not occur more than once or twice per subject at max (i.e., no more than a maximum of two trials per subject should be affected by this). The experimenter was trained before the beginning of data collection, to ensure she was able to systematically code vocal responses within 400 ms.

2 Note that repeating the entire mapping means two consecutive trials with the same combination of stimulus modality and response modality, like auditory-vocal, auditory-vocal. In contrast, auditory-vocal, visual-vocal would be a response-modality repetition, but not a mapping repetition.

3 Note that differences in the patterns of findings between Fintor et al.’s (2020) study and ours may also be in part attributable to the difference between using spatial codes (such as in Fintor et al., 2020) and verbal codes (such as in the present study) in the context of modality-compatibility research (see also Friedgen et al., 2021). In terms of working memory, a verbal stimulus might activate codes residing in the phonological loop, whereas a spatial stimulus might activate codes residing in the visuospatial sketchpad. In the previous study by Friedgen et al. (2021), using verbal stimuli increased the modality-compatibility effect on mixing costs, suggesting a higher load on working memory is required to maintain two modality-incompatible task sets in an activated state, compared to two modality-compatible task sets, when verbal processing codes are involved, as opposed to when spatial processing codes are involved. In Friedgen et al. (2021), we attributed this to a strengthening of the auditory-vocal coupling with verbal processing codes, compared to spatial processing codes.

## Declarations

### Open Practices Statement

The data for all experiments are available at https://osf.io/q7e4p/?view_only=ca0ec13ed2074077a412d33c79be3153. Neither of the experiments was preregistered.
